# Detection of Lettuce Discoloration Using Hyperspectral Reflectance Imaging

**DOI:** 10.3390/s151129511

**Published:** 2015-11-20

**Authors:** Changyeun Mo, Giyoung Kim, Jongguk Lim, Moon S. Kim, Hyunjeong Cho, Byoung-Kwan Cho

**Affiliations:** 1 National Institute of Agricultural Science, Rural Development Administration, 310 Nonsaengmyeong-ro, Wansan-gu, Jeonju-si, Jeollabuk-do 54875, Korea; E-Mails: cymoh100@korea.kr (C.M.); giyoung@korea.kr (G.K.); limjg@korea.kr (J.L.); 2 Environmental Microbiology and Food Safety Laboratory, BARC-East, Agricultural Research Service, US Department of Agriculture, 10300 Baltimore Avenue, Beltsville, MD 20705, USA; E-Mail: Moon.Kim@ars.usda.gov; 3 Experiment & Research Institute, National Agricultural Products Quality Management Service, 141 Yongjeon-ro, Gimcheon-si, Gyeongsangbuk-do 39660, Korea; E-Mail: hjcho201@korea.kr; 4 Department of Biosystems Machinery Engineering, College of Agricultural and Life Science, Chungnam National University, 99 Daehak-ro, Yuseong-gu, Daejeon 34134, Korea; E-Mail: chobk@cnu.ac.kr

**Keywords:** hyperspectral imaging, multispectral imaging, lettuce, discoloration, image processing

## Abstract

Rapid visible/near-infrared (VNIR) hyperspectral imaging methods, employing both a single waveband algorithm and multi-spectral algorithms, were developed in order to discrimination between sound and discolored lettuce. Reflectance spectra for sound and discolored lettuce surfaces were extracted from hyperspectral reflectance images obtained in the 400–1000 nm wavelength range. The optimal wavebands for discriminating between discolored and sound lettuce surfaces were determined using one-way analysis of variance. Multi-spectral imaging algorithms developed using ratio and subtraction functions resulted in enhanced classification accuracy of above 99.9% for discolored and sound areas on both adaxial and abaxial lettuce surfaces. Ratio imaging (RI) and subtraction imaging (SI) algorithms at wavelengths of 552/701 nm and 557–701 nm, respectively, exhibited better classification performances compared to results obtained for all possible two-waveband combinations. These results suggest that hyperspectral reflectance imaging techniques can potentially be used to discriminate between discolored and sound fresh-cut lettuce.

## 1. Introduction

The commercial market for fresh-cut agricultural products in Korea was worth $7000 million in 2012 and is growing every year. Fresh-cut products are divided into three types: lettuce, salad, and other fresh-cut food. The demand for lettuce, which accounts for one of the largest portions of fresh-cut agricultural products, is constantly increasing. In the last 10 years, lettuce production did not show a significant increase and its price increased more than six-fold in Korea. Accordingly, minimizing the loss and sorting high-quality raw lettuce has become ever more important. Browning is an important factor in terms of quality loss during post-harvest storage of lettuce heads and minimally processed lettuce [1,2]. This browning is mainly due to the presence of the polyphenol oxidase enzyme (PPO, enzyme number: EC 1.14.18.1), a mixed function oxidase that first catalyzes the hydroxylation of monophenols to o-diphenols, and then the o-quinones of colorless o-diphenols to highly colored o-quinones [3,4]. Damage generally induces an increase in phenylalanine ammonia-lyse (PAL, EC 4.3.1.5) activity and increased phenolic metabolism in plant tissues [5,6]. In addition, damage induces cellular decompartmentalization, which allows the mixing of phenolic substrates and PPO, leading to the development of browning [4,7].

The appearance of leafy vegetables is very important in terms of the consumer [8]. Defects such as browning occur during storage, with foreign materials in lettuce, such as worms and slugs, also causing consumer complaints, although the complete removal of these defects during the cleaning process is difficult. Surface defects such as browning of fresh-cut lettuce are inspected by operators using the naked eye. Therefore, non-destructive, rapid techniques for the evaluation of surface defects on fresh-cut lettuce are required.

Various studies aiming to determine external and internal quality and analyze the defects of agricultural products such as vegetables and fruits have been performed using near-infrared (NIR) spectroscopy, machine vision, multispectral imaging, and hyperspectral imaging techniques [9,10,11,12,13]. The conventional image system is incapable in inspecting specimens with similar color and discriminate complex objectives [14]. Hyperspectral imaging is a promising nondestructive measurement technique [15], which involves the acquisition of spatial and spectral information simultaneously for each pixel in a sample image. Hence, hyperspectral imaging can provide physical and chemical information beyond that provided by the simple optical R/G/B regions used by conventional imaging systems. This technique can also be employed in order to determine the subtle physical and chemical characteristics of an object, and to visualize the spatial distributions of its chemical components.

Hyperspectral imaging and multispectral technology has been examined as a potential method for food defects and safety assessment, such as in the detection of fecal contamination and defects on apples and defects on tomatoes, and the detection of microbial contamination such as bacterial biofilms on a food-processing surface [16,17,18,19,20]. A method for estimating the tissue damage during the processing of fresh-cut vegetables was developed using optical imaging technology [21]. A multispectral fluorescence imaging technique was employed in order to develop two-waveband algorithms for the detection of bovine fecal contaminants on the surface of romaine lettuce and baby spinach leaves [22]. Hyperspectral imaging is used to find two- or three-wavebands to detect the defects. In order to design a low-cost multispectral camera with the two- or three-wavebands optimized for quality measurement of lettuce, the hyperspectral imaging technique can be used to find two- or three-waveband to detect the quality such as the defects.

In this study, a rapid method for detecting discoloration on lettuce surfaces was developed using a near-infrared hyperspectral imaging technique. Hyperspectral reflectance imaging analysis was used in order to determine the appropriate multispectral bands for use in detecting surface defects on lettuce. The objectives of this study were to determine the most significant wavelengths for defect evaluation and to develop multispectral imaging algorithms with the function of ratio and subtraction for detecting discoloration on fresh-cut lettuce, to be used in online inspection applications in fresh-cut vegetable processing plants.

## 2. Experimental Section

### 2.1. Materials

Lettuce (*Lactuca sativa*) produced in the southern part of Korea in 2014 was purchased and used in the experiments. The samples were stored in a refrigerator at 2 °C for 4 day after harvesting as the optimum temperature for storage of lettuce is 0~2 °C [23], and lettuce leaves that exhibited discolored areas with browning and defects were taken for use in the experiments. A total of 60 discolored and 60 sound lettuce leaves were cut in order to produce samples with dimensions of 5 cm × 5 cm. Hyperspectral images of each adaxial and abaxial surface of the samples were obtained in order to develop methods that can be used on both surfaces of a leaf sample, immediately after cutting. The samples were equilibrated at room temperature before cutting, in order to obtain the hyperspectral images. There were 30 calibration samples (Set A) used for algorithm development and 30 validation samples (Set B) used to test the algorithms.

### 2.2. Hyperspectral Imaging System

**Figure 1:**
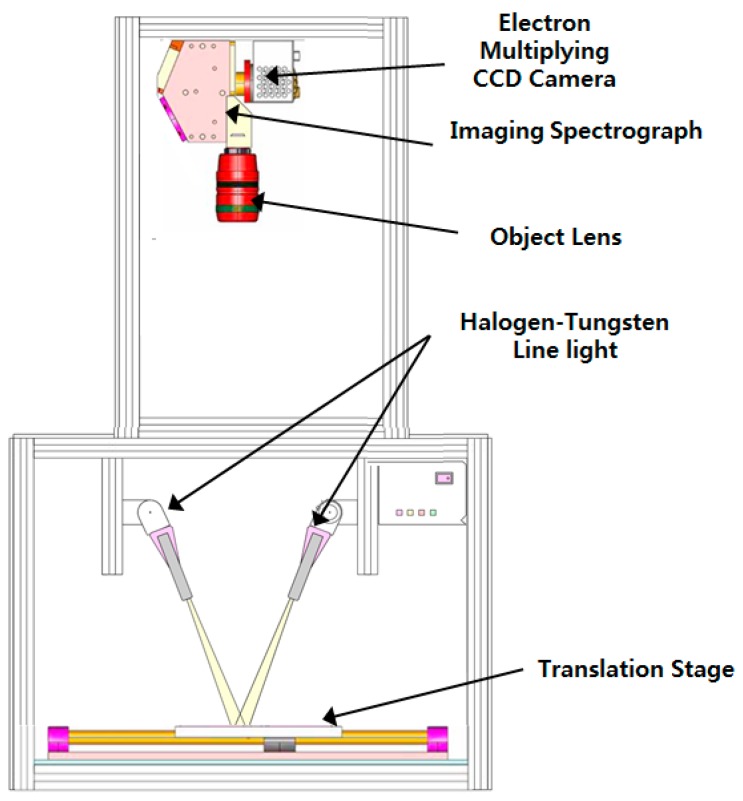
Schematic representation of the hyperspectral reflectance imaging system.

Figure 1 shows a schematic diagram of the hyperspectral line-scan imaging system equipped with the critical components. This imaging system was composed of a low-light sensitive electron multiplying charge-coupled device (EMCCD) camera (MegaLuca, Andor Technology Inc., Belfast, Northern Ireland), an imaging spectrograph (VNIR Hyperspec, HeadwallPhotonics Inc., Fitchburg, MA, USA), a Schneider-Kreuznach Xenoplan 1.4/23 C-mount lens (Schneider Optics, Hauppauge, NY, USA), and a pair of light sources. The EMCCD camera with a resolution of 1002 vertical and 1004 horizontal pixels was thermo-electrically cooled to a temperature of −20 °C using a two-stage Peltier device. The imaging spectrograph with a 25-µm slit was attached, together with a C-mount lens with focus adjustment and an aperture diaphragm. Passing through the 25 µm × 18 mm (width × length) aperture slit, light from the scanned line of a field-of-view (FOV) was dispersed by the dispersive grating and projected onto the EMCCD. A two-dimensional image with the spatial dimension along the horizontal axis and the spectral dimension along the vertical axis of the EMCCD was created. In the case of reflectance imaging, the spectral image amplified by the EMCCD camera was captured only in the wavelength range from 400 to 1000 nm.

### 2.3. Hyperspectral Image Spectra Acquisition

Twelve individual sets of hyperspectral images were obtained for lettuce, 6 for the adaxial and 6 for the abaxial surfaces, each set consisting of a 5 × 2 cut leaf arrangement on a lined tray. Line-scan images were captured for exposure times of 6 ms at 0.5 mm intervals, and each set consisted of 600 line scans corresponding to 600 × 502 pixels per spectral band. The hyperspectral images were composed of a total of 125 spectral bands in the range from approximate 400 to 1000 nm, with a waveband interval of 4.8 nm.

The dark reference images were acquired in the absence of a light source in order to correct for the noise incurred by the EMCCD camera. The white reference images were obtained using a 99% diffuse reflectance standard (Spectralon™, SRT-99-120, Labsphere, NH, USA) in order to calibrate the intensity of the light source for each vertical pixel. The dark and white reference images were used in order to convert raw reflectance images of lettuce into corrected reflectance images, according to the following equation:

(1) $$I_{\mathrm{reflectance}}\left(i\right)\;=\;\frac{{\text{I}}_{\text{raw}}\;\left(\text{i}\right)-{\text{ I}}_{\text{dark}}}{{\text{I}}_{\text{white}}\;-{\text{ I}}_{\text{dark}}}$$

where I_reflectance_ is the corrected reflectance image at the i^th^ wavelength, I_raw_ is the raw hyperspectral image at the i^th^ wavelength, I_dark_ is the dark reference image at the i^th^ wavelength, and I_white_ is the white reference image at the i^th^ wavelength.

In terms of the hyperspectral images corrected, the reflectance spectra of the pixels composed of sound and discolored areas of a lettuce surface were extracted and used to calculate an average reflectance spectrum.

The degree of discoloration of the lettuce was divided into three levels (slight, intermediate and severe) based on the visual inspection of two trained human inspectors. The area where the discoloration degree is greater than the minimum level with light pink color was determined to be discolored area. Note that there has not been a standard reference for discoloration of lettuce and no imaging instrument exceeds the performance of inspection by human eyes. Average spectra were obtained by manual selection of sound and discolored areas. The region of interest (ROI) was selected in a polygonal shape to freely select indeterminate shapes of sound and discolored areas.

In order to classify sound and discolored areas on lettuce surfaces, simple single waveband and two-waveband multispectral imaging algorithms were developed. The single waveband imaging (SWI) algorithm was developed using the reflectance value (R) of a single waveband. In the case of two wavebands, two-waveband ratio imaging (RI) and subtraction imaging (SI) algorithms were developed using the ratio image (R_a/b_) and subtraction image (S_a-b_) of reflectance values at two wavebands, respectively.

In order to determine the optimal waveband pairs for use in the detection of discolored and sound areas in the samples employed in this study, one-way analysis of variance (ANOVA) was performed using 23,599 spectra extracted within the region of interest (ROI) from sound and discolored areas on 30 abaxial and adaxial lettuces from Set A were used for calibration. In order to select the optimal wavebands for detecting discoloration, the ANOVA F-values were calculated for the single waveband, two-waveband ratio, and two-waveband subtraction approaches for all possible two-waveband combinations. Using these optimal wavebands, each of these three types of algorithms were developed for use with the average spectrum and pixel spectra. The optimal global threshold was then determined at the highest classification accuracy. The validation of the developed algorithms was conducted using three types of average spectrum, pixel spectra, and waveband images from 30 Set B samples. The MATLAB software (version 7.0.4, the Mathworks, Natick, MA, USA) was used to extract and analyze the hyperspectral image data.

## 3. Results and Discussion

### 3.1. Spectral Characteristics of Sound and Discolored Lettuce

**Figure 2:**
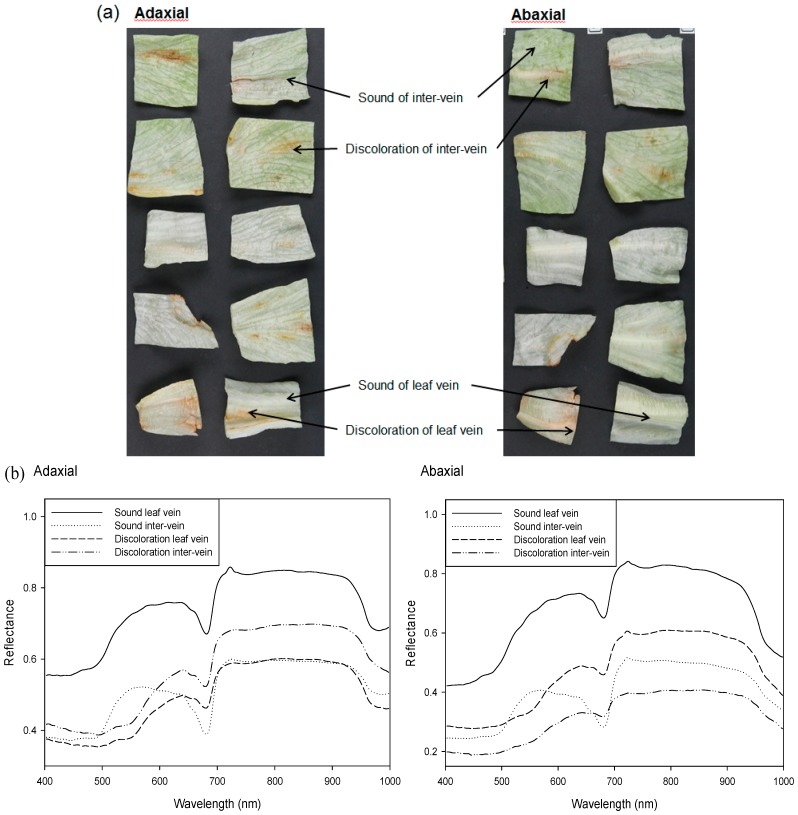
(**a**) Images of lettuce leaves and (**b**) representative reflectance spectra of discolored and sound areas on the veins and inter-vein regions of adaxial and abaxial lettuce leaf surfaces.

Representative spectra, extracted from corrected hyperspectral lettuce images that included discolored and sound areas containing primary veins, secondary veins, and leaf interveins, were measured in the wavelength range from 400 to 1000 nm (Figure 2). These features are also indicated on the reflectance images of the adaxial and abaxial leaf surfaces. Each spectral image depicts a mean for the spectra obtained for 60–100 individual pixels of interest. The reflectances of sound areas of leaf vein and intervein for both adaxial and abaxial surfaces in the range from 483 to 540 nm (with a blue–green color), which included the absorbance wavelength of carotenoids with the inhibition characteristics of an enzymatic browning reaction, increased more sharply than those of the discolored areas [24]. However, the reflectance of discolored areas of leaf vein and intervein for both surfaces in the range from 540 to 640 nm (yellow–orange color), which included the wavelength related to enzymatic browning of anthocyanin, increased more sharply than those of the sound areas [25]. Moreover, the reflectance of the sound lettuce area in the range from 640 to 685 nm exhibited a sharp reduction compared to the reflectance of the discolored area. The dominant chlorophyll absorption feature in the spectra was observed at approximately 681 nm [26].

### 3.2. Algorithm for Distinguishing Discoloration on Lettuce Using Spectra

**Figure 3:**
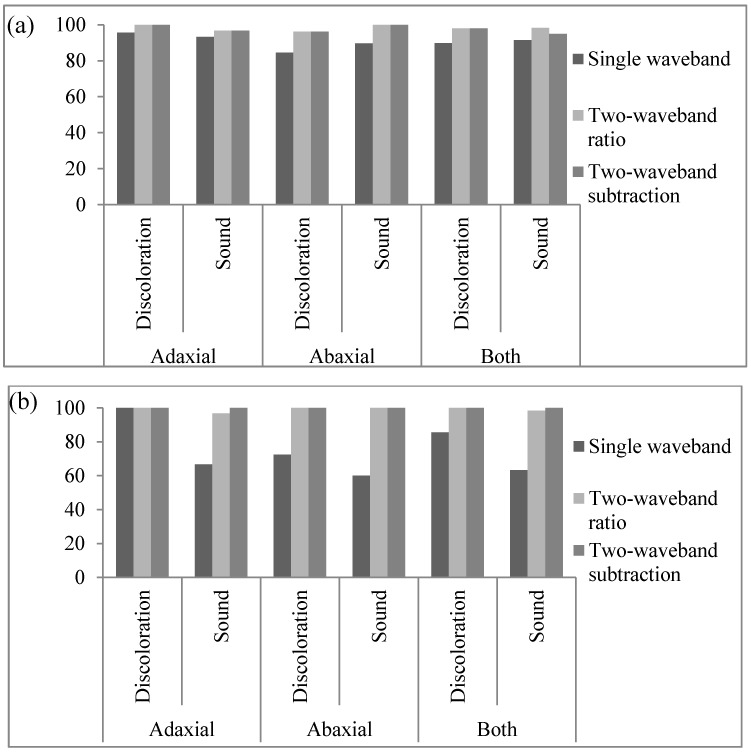
Classification accuracy of adaxial surface, abaxial surface and both surfaces using a single waveband algorithm and a combination of a two-waveband algorithm and average spectra: (**a**) calibration (Set A) and (**b**) validation (Set B).

**Figure 4:**
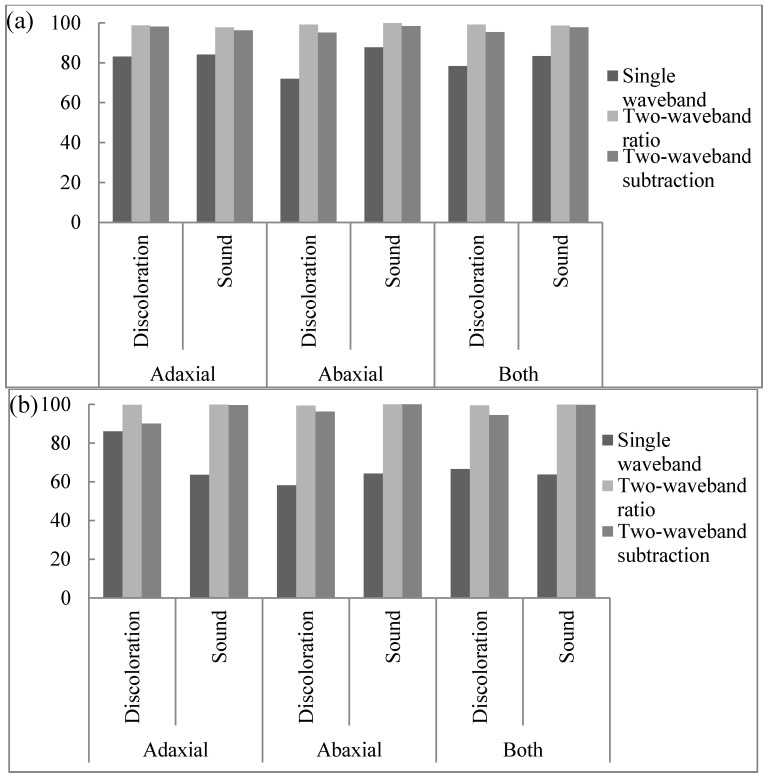
Classification accuracy of adaxial surface, abaxial surface and both surfaces using a single waveband algorithm and a combination of a two-waveband algorithm and pixel spectra: (**a**) calibration (Set A) and (**b**) validation (Set B).

Algorithms using single bands or the function of ratio and subtraction used to detect discoloration on the lettuce surface were developed using hyperspectral reflectance spectra. Discoloration detection methods using single and multi-waveband spectra were investigated using one-way ANOVA. The discrimination results and classification values of three algorithms in terms of discoloration detection capacity using an average spectrum and the pixel spectra for sound and discolored areas on the abaxial and adaxial lettuce surface are shown in Table 1, Table 2, Figure 3 and Figure 4.

**Table 1:** Discoloration discrimination results using a single waveband algorithm and a combination of a two-waveband algorithm and average spectra.

	Type of Surface		Calibration (Set A)			Validation (Set B)		
			Single Waveband	Two-Waveband Ratio	Two-Waveband Subtraction	Single Waveband	Two-Waveband Ratio	Two-Waveband Subtraction
		Optimal Wavebands	547 nm	552 nm, 701 nm	557 nm, 701 nm	547 nm	552 nm, 701 nm	557 nm, 701 nm
No. of total sample	Adaxial	Discoloration	23			26		
		Sound	30			30		
	Abaxial	Discoloration	26			29		
		Sound	29			30		
^1)^ CA (%)	Adaxial	^2)^ CV	0.44~0.45	0.85–0.89	−0.086 ~ −0.074	0.44	0.85	−0.086 ~ −0.084
		Discoloration	95.7	>99.9	>99.9	>99.9	>99.9	>99.9
		Sound	93.3	96.7	96.7	66.7	96.7	>99.9
	Abaxial	^2)^ CV	0.44	0.79–0.85	−0.098	0.44	0.85	−0.086 ~ −0.084
		Discoloration	84.6	96.2	96.2	72.4	>99.9	>99.9
		Sound	89.7	>99.9	>99.9	60.0	>99.9	>99.9
	Both	^2)^ CV	0.44	0.85	−0.086 ~ −0.084	0.44	0.85	−0.086 ~ −0.084
		Discoloration	89.8	98.0	98.0	85.5	>99.9	>99.9
		Sound	91.5	98.3	94.9	63.3	98.3	>99.9

Note: ^1)^ CA, classification accuracy; ^2)^ CV, classification value.

**Table 2:** Discoloration discrimination results using a single waveband algorithm and a combination of a two-waveband algorithm and pixel spectra.

	Type of Surface		Calibration (Set A)			Validation (Set B)		
			Single Waveband	Two-Waveband Ratio	Two-Waveband Subtraction	Single Waveband	Two-Waveband Ratio	Two-Waveband Subtraction
		Optimal Wavebands	547 nm	552 nm, 701 nm	557 nm, 701 nm	547 nm	552 nm, 701 nm	557 nm, 701 nm
No. of total pixels	Adaxial	Discoloration	2694			1213		
		Sound	6919			16,917		
	Abaxial	Discoloration	3882			2836		
		Sound	10,147			4854		
^1)^ CA (%)	Adaxial	^2)^ CV	0.44	0.78	−0.089	0.43	0.81	−0.106
		Discoloration	83.1	98.8	98.1	86.0	99.7	90.0
		Sound	84.1	97.8	96.3	63.6	99.9	99.6
	Abaxial	^2)^ CV	0.41	0.81	−0.106	0.43	0.81	−0.106
		Discoloration	72.0	99.1	95.1	58.2	99.4	96.2
		Sound	87.7	99.9	98.4	64.2	>99.9	>99.9
	Both	^2)^ CV	0.43	0.81	−0.106	0.43	0.81	−0.106
		Discoloration	78.4	99.1	95.4	66.5	99.5	94.4
		Sound	83.4	98.7	97.8	63.7	99.9	99.7

Note: ^1)^ CA, classification accuracy; ^2)^ CV, classification value.

#### 3.2.1. Single Waveband Algorithm

**Figure 5:**
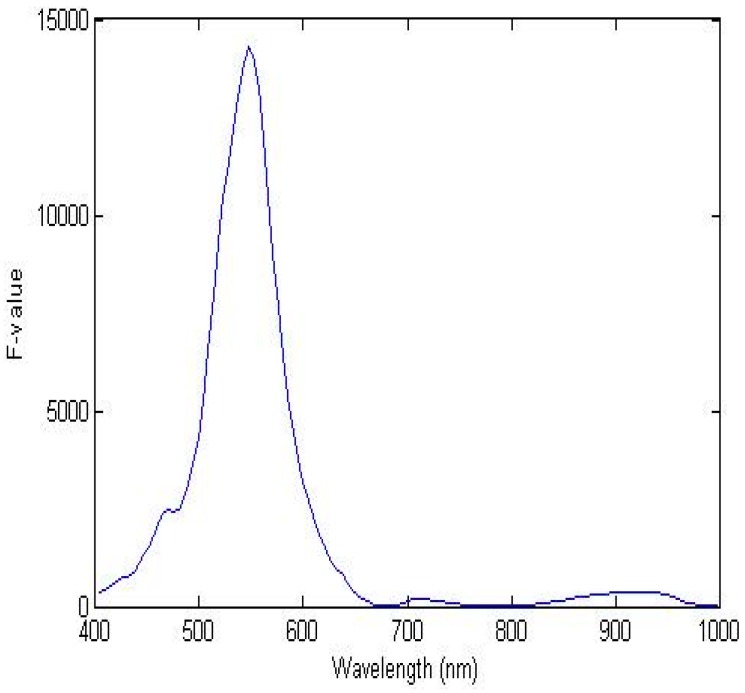
One-way ANOVA results for classifying sound and discolored areas of lettuce using the single waveband algorithm in the wavelength range from 400 to 1000 nm.

The single waveband algorithm for detecting lettuce discoloration was developed using one waveband. Figure 5 shows the F-value of each waveband from the ANOVA results for distinguishing discolored and sound areas on lettuce samples, using one waveband in the range from 400 to 1000 nm. The F-values for discolored and sound portions of lettuce were calculated for all 125 wavebands in the 400–1000 nm region in order to determine the most appropriate waveband. The optimal waveband with the highest F-value (F) of 14,299.3 was at 547 nm, which is on the boundary between green and yellow, and is the waveband related to cellular browning of anthocyanin [25]. The reflectance intensity of sound lettuce at 547 nm was lower than that of the discolored lettuce, as shown in Figure 2b. Degradation of anthocyanin pigments has been reported as a possible mechanism for color loss in strawberries [27]. The peaks in the F-value were at approximately 470 nm and 710 nm, which are related to carotenoids and chlorophyll, respectively [26,28].

**Figure 6:**
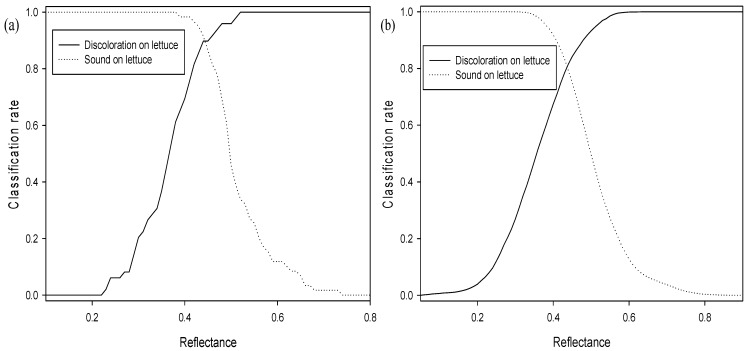
Classification rates for sound and discolored areas on both adaxial and abaxial surfaces using the reflectance of a 547 nm single waveband with calibration samples: (**a**) average spectrum and (**b**) pixel spectra.

As shown in Figure 6a,b, the classification accuracies of sound and discolored areas on both adaxial and abaxial lettuce surfaces were indicated by the reflectance of a 547 nm single band for the average spectrum and the pixel spectra of the calibration sample (Set A). The threshold values for the abaxial and adaxial lettuce surfaces with the best classification using the average spectrum were 0.44 and 0.42, respectively, as shown in Table 1 and Figure 4a. The classification accuracies of the abaxial surface were above 99.9% for discolored areas and 66.7% for sound areas, which were higher than those of the adaxial surface (Figure 3b). The classification accuracies of discolored and sound areas on both the adaxial and abaxial surfaces were 78.2% and 73.3%, respectively. The developed algorithm was verified using the validation samples (Set B). The validation results for this algorithm on the average spectrum, using classification values determined for detecting surface defects of Set A samples, showed that the classification accuracies for discolored and sound areas on abaxial and adaxial surfaces were improved by 89.8% and 91.5%, respectively. The classification accuracies on abaxial surfaces for validation samples (Set B) were higher than those on adaxial surfaces.

The single waveband algorithm used to discriminate between sound and discolored areas of lettuce was developed using pixel spectra. The discrimination accuracies of the sound and discolored areas on both surfaces were 78.3% and 83.4%, respectively, lower than those using average spectra (Table 2, Figure 6b). This may be attributed to the noise reduction for each pixel spectrum caused by averaging the total pixel spectrum. Threshold values for pixel spectra were also shifted to lower values than those of average spectra, and the threshold values of both the average and pixel spectra were higher for abaxial surfaces than for adaxial surfaces. The validation results of this algorithm for sound and discolored areas on both surfaces were 66.5% and 63.7%, respectively (Figure 4b). The classification performances of the both sound and discolored groups on both surfaces using the pixel spectra for calibration samples (Set A) were higher than those for validation samples (Set B) given in Table 2.

#### 3.2.2. Two-Waveband Ratio Algorithm

**Figure 7:**
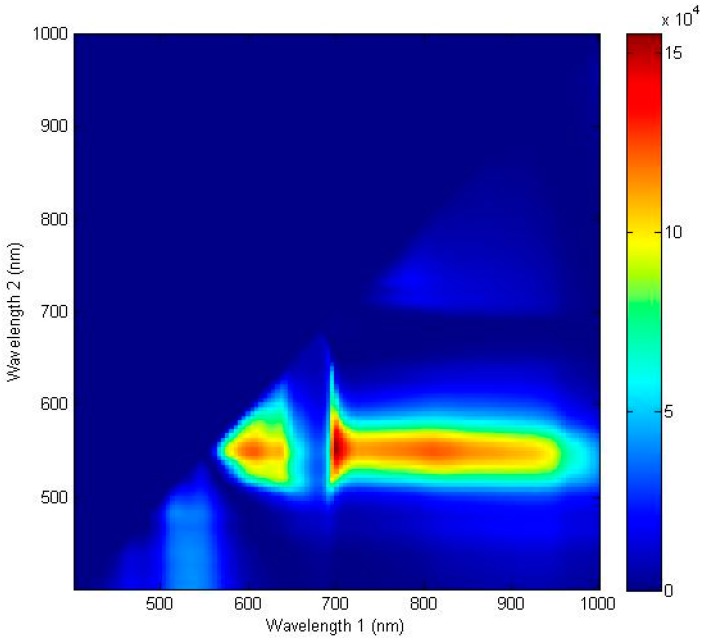
Waveband selection for the ratio spectra and imaging algorithm. One-way ANOVA results for classifying sound and discolored areas of the lettuce surface using the ratio of two wavebands in the wavelength range from 400 to 1000 nm.

The ratio discrimination algorithm for predicting the discoloration of lettuce surfaces was developed using two reflectance wavebands. Figure 7 shows the F-values calculated by one-way ANOVA for all possible two waveband ratios for the spectra of the discolored and sound areas described by a contour plot. The contour image indicated the most effective spectral wavebands for discriminating between discolored and sound surfaces. A larger F-value designated a more statistically significant mean separation between the two groups. The optimal wavebands of the ratio were 552 nm and 701 nm with an F-value of 155,522.3. These wavebands near the F-value peak of a single waveband are associated with the characteristics of anthocyanin and chlorophyll [29,30,31]. The results indicated that the F-values of the two-waveband ratio combinations for detecting discoloration on a lettuce surface produced were higher than those of the single waveband. These two peaks were close to the F-value peaks of the single waveband with a high correlation used to discriminate between the discolored and sound lettuce areas, as shown in Figure 5. As shown in Figure 6b, the ratio values (R_552/701_) of the discolored lettuce areas were higher than those of the sound areas.

**Figure 8:**
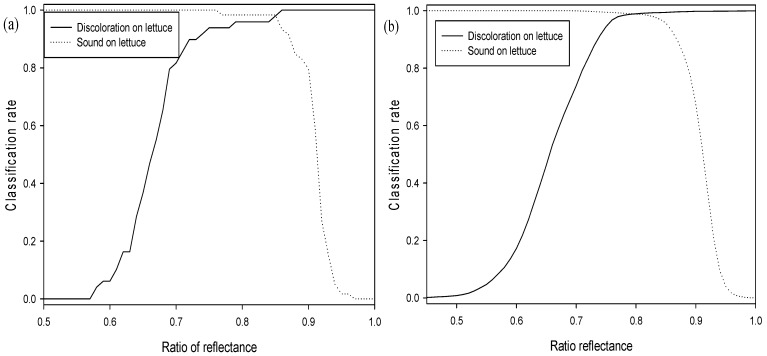
Classification rate results for sound and discolored areas on both adaxial and abaxial surfaces using the ratio of two wavebands (R_552/701_): (**a**) average spectrum and (**b**) pixel spectra.

Figure 8a,b shows the correlation between the classification accuracy of sound and discolored areas on both adaxial and abaxial lettuce surfaces, the value of the ratio between 552 nm and 701 nm for an average spectrum, and the pixel spectra for the calibration samples (Set A). Threshold values of adaxial and abaxial surfaces (Set A) from 0.79 to 0.83 and from 0.82 to 0.83, respectively, gave the best classification results for the average spectrum (Table 1, Figure 8a). The classification accuracies of discolored and sound areas on both surfaces were above 99.9% higher than those obtained using the single waveband algorithm (Figure 3b). The results using this algorithm were validated using the validation sample (Set B), using a classification value determined for detecting defects on both Set A surfaces, with classification accuracies for discolored and sound areas on adaxial and abaxial surfaces of 98.0% and 98.3%, respectively. The classification accuracies of the adaxial and abaxial surfaces for the validation samples (Set B) were similar to these values, and perhaps the difference between both surfaces was reduced due to the ratio treatment.

The two-waveband ratio algorithm used to predict the presence of sound and discolored areas of lettuce surfaces were developed using pixel spectra. The classification performances of sound and discolored areas on both surfaces of the pixel spectra were 99.1% and 98.7%, respectively. These were lower than those values obtained using average spectra for the calibration samples (Set A), but higher than those obtained using average spectra for the Set B validation samples (Table 2, Figure 8b). This may be attributed to a reduction in the noise of each pixel spectrum caused by taking the average of the total pixel spectrum. The threshold values for detecting discoloration in the pixel spectra (Figure 8b) were also shifted to lower values than those of the average spectrum (Figure 8a). The threshold values for both the average spectrum and pixel spectra for abaxial surfaces were higher than those of the adaxial surfaces. The validation results for this algorithm for sound and discolored areas on both surfaces indicated accuracies of 99.5% and 99.9%, respectively (Figure 4b). The two-waveband R_552/701_ ratio algorithm was able to detect lettuce discoloration.

#### 3.2.3. Two-Waveband Subtraction Algorithm

**Figure 9:**
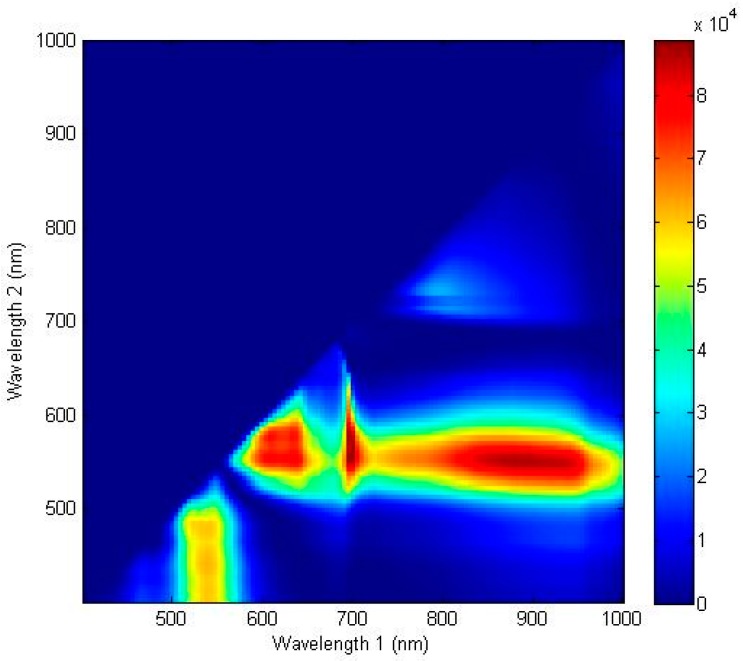
Waveband selection for the subtraction spectra and imaging algorithm. One-way ANOVA results for classifying sound and discolored areas of lettuce using the subtraction of two wavebands in the wavelength range from 400 to 1000 nm.

**Figure 10:**
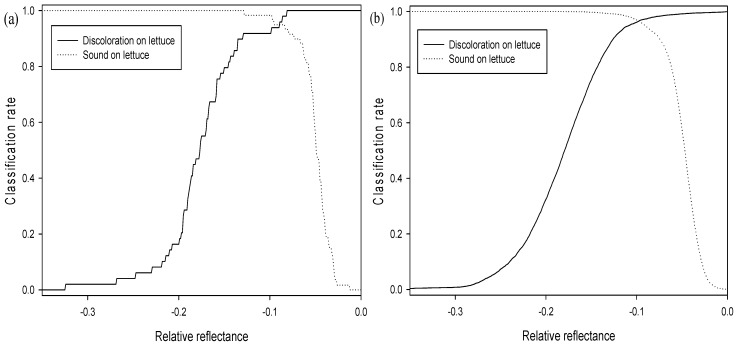
Classification rate results for the sound and discolored areas on both adaxial and abaxial surfaces using the subtraction of two wavebands (S_557-701_): (**a**) average spectrum and (**b**) pixel spectra.

The two-waveband subtraction algorithm developed in order to predict lettuce discoloration employed two reflectance wavebands. The one-way ANOVA F-values for all possible two waveband subtractions (125 × 125) for the sound and discolored areas are illustrated in the contour plot in Figure 9. The discoloration of lettuce as correlated to reflectance subtraction for the waveband pairs is shown in Figure 9. The F-values for distinguishing between discolored and sound lettuce surfaces were calculated for all 58 × 58 wavebands in the 400–1000 nm region in order to find the best combination for two-waveband subtraction. The subtraction image at wavebands of 557 nm and 701 nm (S_557-701_) exhibited the highest F-value of 88756.0. The wavebands near the F-value peak of the single waveband are associated with the characteristics of anthocyanin and chlorophyll [25,26,27]. The F-value obtained when subtracting between these two wavebands in order to classify discolored lettuce was greater than that of the single waveband. Figure 10b indicates that the two-waveband subtraction (S_557-701_) values for sound lettuce were lower than those of discolored lettuce.

The relation between the classification rates for sound and discolored lettuce, and the value of the subtraction between the 557 nm and 701 nm wavebands are given in Figure 10 for the average spectrum and pixel spectra on both adaxial and abaxial calibration sample surfaces (Set A). The threshold values for adaxial and abaxial Set A sample surfaces for the best classification values obtained using the average spectrum were from −0.086 to −0.074 and −0.098, respectively (Figure 10a). The classification accuracies for discolored and sound areas on both surfaces were 98.0% and 94.9%, respectively, and showed an improvement compared to those obtained using the single waveband algorithm (Figure 3a). The classification performance for discriminating discoloration on the adaxial surface and sound areas on the abaxial surface exceeded that of sound areas on the abaxial surfaces and discoloration areas on the adaxial surfaces, respectively. The validation results for this algorithm using the validation sample (Set B) and a classification value determined for the calibration samples (Set A) show that the classification accuracies for discolored and sound areas on both surfaces increased by above 99.9% (Figure 3b). As the two-waveband subtraction algorithm was more accurate than the single waveband algorithm, the difference in the classification accuracies for the adaxial and abaxial surfaces of the validation samples (Set B) was reduced.

The two-waveband subtraction algorithm for predicting the presence of sound and discolored areas of lettuce was developed using pixel spectra (Table 2). The classification accuracies for sound and discolored areas on both surfaces of the calibration samples (Set A) were 95.4% and 97.8%, respectively (Figure 10b). The validation results for this algorithm for sound and discolored areas on both surfaces were 94.4% and 99.7%, respectively (Figure 4b). The threshold values for the pixel spectra (Figure 10b) were shifted to lower values than those of the average spectra (Figure 10a). The classification performances of calibration Set A for discolored areas on both surfaces using the pixel spectra were higher than those of validation Set B (Table 2). The S_557-701_ two-waveband subtraction algorithm indicated a capacity for detecting lettuce discoloration.

The classification results for the single waveband algorithm showed that the calibration set for both the average spectrum and the pixel spectra exhibited higher accuracy than the validation set. The classification results for the two-waveband ratio and subtraction algorithms, however, indicated that the prediction set for both types of extraction spectrum exhibited higher accuracy than the calibration set. The classification performances of both the two-waveband ratio and two-waveband subtraction algorithms were improved when these algorithms were applied to the validation samples. In case of two-waveband ratio and two-waveband subtraction algorithms, detection of discoloration on adaxial surfaces and sound areas on abaxial surfaces exhibited better performances. The best threshold values obtained for adaxial surfaces for the three types of algorithms under examination using the average spectrum and pixel spectra were lower than those of abaxial surfaces, except in the case of the two-waveband ratio algorithm when using the pixel spectra.

### 3.3. Development of Imaging Algorithms for Discoloration Discrimination

The algorithms for predicting discoloration on lettuce were developed using hyperspectral reflectance imaging. Discoloration discrimination methods were examined using single and multi-waveband images, determined using ANOVA analysis. Table 3 shows the discoloration discrimination results for single waveband, two-waveband ratio, and two-waveband subtraction imaging algorithms with validation samples (Set B) using the classification values for pixel spectra shown in Table 2.

#### 3.3.1. Single Waveband Imaging (SWI) Algorithm

**Figure 11:**
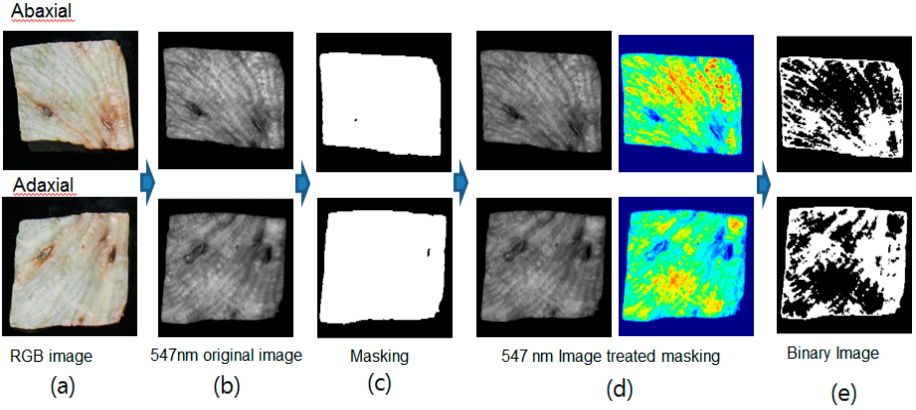
Illustration of the image processing sequence using a single waveband: (**a**) sample image; (**b**) grayscale I_547_ image; (**c**) masking images obtained by applying a threshold of 0.07 reflectance to an I_547_ image; (**d**) grayscale and color images of I_547_ after masking; and (**e**) resulting detection images after the application of a 0.44 threshold.

Figure 11 shows a sequence of representative images processed using single waveband images in order to classify discolored areas. The images for the 547 nm single waveband (I_547_ in Figure 11b) were converted into binary images (Figure 11c), where reflectance values greater than 0.07 were given a value of “1” and those less than 0.07 were given a value of “0”. The backgrounds were then eliminated and a masking image was produced in order to select only lettuce image areas. The 547 nm waveband image (Figure 11d) was created by applying the masking image and then converted into the binary form using a threshold reflectance value of 0.44 (Figure 11e). As shown in Figure 7, which indicates the classification rate of sound and discolored areas using the SWI algorithm for both adaxial and abaxial lettuce surfaces, this threshold value yielded the best accuracy for discriminating between sound and discolored areas when using the calibration samples (Set A). If the pixel number of positive area is more than 4, this area was considered to the discoloration area. The positive area in the binary image represented the discolored areas of the lettuce.

#### 3.3.2. Ratio Imaging (RI) Algorithm

**Figure 12:**
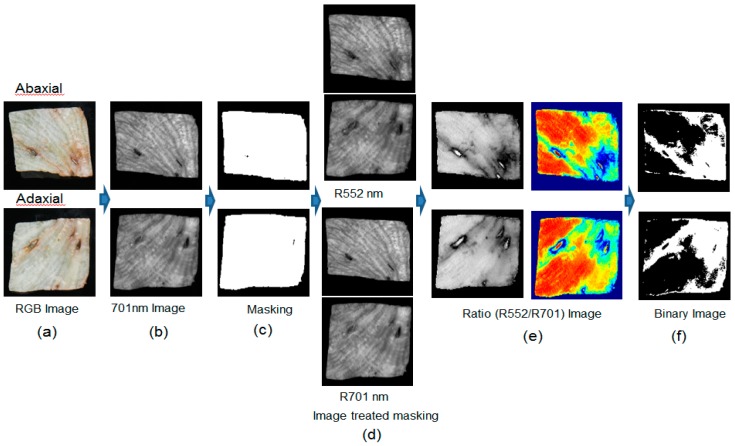
Illustration of image processing sequence for the RI algorithm using two wavebands: (**a**) sample images; (**b**) grayscale I_701_ images; (**c**) masking images obtained by applying a threshold of 0.10 reflectance to an I_701_ image; (**d**) I_552_ and I_701_ images after masking; (**e**) the grayscale and color ratio images of I_552_ and I_701_ after masking; and (**f**) resulting detection images after the application of a 0.82 threshold.

Figure 12 shows a sequence of images corresponding to the image processing method used to distinguish the discolored areas of lettuce samples using the two-waveband ratio imaging (RI) algorithm. Figure 12a shows the RGB images of the adaxial and abaxial lettuce samples obtained by conventional digital camera with one image. The 701 nm image with the greatest difference in intensity between the background and the lettuce for the two wavebands was selected as the masking image (Figure 12b). The binary image using the 701 nm image for masking was created following the same method as was described for the case of the SWI algorithm (Figure 12c). A threshold value of 0.1 was obtained using the Otsu threshold method. The ratio images for 552 nm and 701 nm (R_552/701_) (Figure 12e) were created using 552 nm and 701 nm images after the application of the masking image (Figure 12d). The ratio images were transformed into binary images using the threshold value (Figure 12f). The classification rate of sound and discolored areas using the two-waveband ratio is shown in Figure 8. The threshold value of 0.82 was determined as yielding the best classification accuracy for the sound and discolored areas using the calibration sample of Set A. If the pixel value of the ratio image was higher than the threshold value of 0.82, the pixel was assigned a value of 1. If the pixel value of the ratio image was lower than the threshold value of 0.82, the pixel value was assigned a value of 0. The positive areas of these binary images represent discoloration.

#### 3.3.3. Two-Band Subtraction Imaging (SI) Algorithm

**Figure 13:**
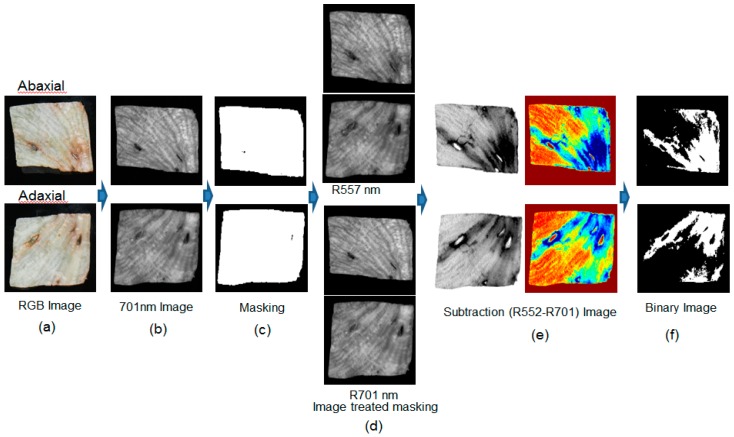
Illustration of image processing sequence for the subtraction imaging (SI) algorithm using two wavebands: (**a**) sample images; (**b**) grayscale I_701_ images; (**c**) masking images obtained by applying a threshold of 0.10 reflectance to the I_701_ image; (**d**) I_557_ and I_701_ images after masking; (**e**) the grayscale and color subtraction images between I_557_ and I_701_ after masking; and (**f**) resulting detection images after the application of a 0.82 threshold.

Figure 13 shows a sequence of images corresponding to the image processing performed in order to classify discolored lettuce using the two-waveband subtraction imaging (SI) algorithm. The 701 nm masking images were created using the same method as was described for the case of the ratio imaging (RI) algorithm (Figure 13b,c). The subtraction images (Figure 13e) were obtained after the 557 nm and 701 nm lettuce images were masked (Figure 13d). The subtraction images were converted into binary images using a threshold value of −0.106, which gave the best classification accuracy (Figure 13f). Figure 8 shows the classification rate of the sound and discolored areas using two-waveband subtraction. If the pixel value of the ratio image was higher than the threshold value of −0.106, the pixel was assigned a value of 1. If the pixel value of the ratio image was lower than the threshold value of −0.106, the pixel value was assigned a value of 0. The positive parts of the binary images show the areas of each lettuce that were identified as being discolored.

#### 3.3.4. Classification Results for the Three Imaging Algorithms

**Figure 14:**
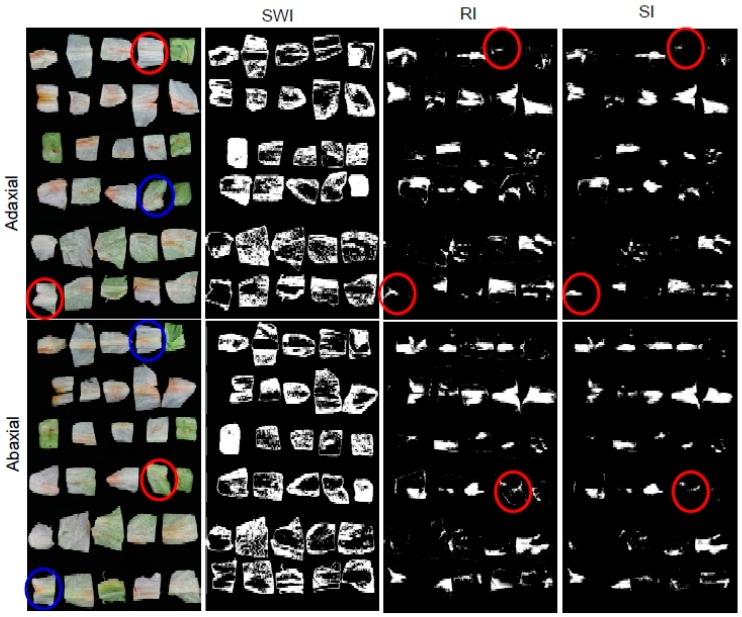
Prediction results for (**a**) an RGB picture of the validation samples; (**b**) SWI algorithm (I_547_); (**c**) RI algorithm (R_552/701_); and (**d**) SI algorithm (S_557-701_) using fresh-cut lettuce samples.

The imaging prediction results of the three algorithms obtained using validation samples are shown in Table 3. Figure 14 shows the discoloration detection results for validation sample B, using the SWI algorithm with a 547 nm image, the RI algorithm with the R_552/701_ ratio image, and the SI algorithm with the S_557-701_ subtraction image. The prediction results for the SWI algorithm indicated that the positive areas of the prediction image, the discoloration, were larger than the discolored areas of the samples (Figure 14b). The prediction accuracies of discoloration area for adaxial and abaxial surfaces were above 46.4% and 42.4%, respectively. The sensitivity, classification accuracy of true positive for the detection of discoloration areas of adaxial and abaxial surfaces, were above 99.9% and 86.2%, respectively. The specificity, true negative rate meaning misclassification of the discoloration area as the sound ones, were 0% for the adaxial and abaxial surfaces. The reason of the low classification accuracy is because the white and green color regions of discoloration areas were recognized as sound surfaces with the single waveband images. The prediction image obtained using the SWI algorithm presented difficulties in distinguishing discoloration from sound areas. The detection of discoloration using the imaging algorithm exhibited better accuracy than that obtained using two types of extraction spectra, but discriminating between sound and discolored areas using single waveband images algorithm affords a lower accuracy than that obtained using two types of extraction spectra.

**Table 3:** The results of discoloration detection using images of lettuce surfaces for three imaging algorithms.

	No. of Samples		CA ^1)^ (%)								
			SWI ^2)^			RI ^3)^			SI ^4)^		
	Discoloration	Sound	SE ^5)^	SP ^6)^	PA ^7)^	SE ^5)^	SP ^6)^	PA ^7)^	SE ^5)^	SP ^6)^	PA ^7)^
Adaxial surface	26	30	>99.9	0	46.4	>99.9	>99.9	>99.9	>99.9	>99.9	>99.9
Abaxial surface	29	30	86.2	0	42.4	>99.9	>99.9	>99.9	>99.9	>99.9	>99.9

Note: ^1)^ CA, classification accuracy; ^2)^ SWI, single-waveband image; ^3)^ RI, two-waveband ratio image; ^4)^ SI, two-waveband subtraction image; ^5)^ SE, sensitivity; ^6)^ SP, specificity; ^7)^ PA, predictive accuracy.

In case of the RI and SI algorithms, the predictive accuracy for detecting discoloration areas was above 99.9% for both adaxial and abaxial surfaces. The sensitivity and the specificity for adaxial and abaxial surfaces were also above 99.9%. As indicated in red in Figure 14c,d, the sound areas of four samples of adaxial surfaces and one sample of an abaxial surface gave false positives that indicated discoloration. The reverse sides of the sound areas of these samples did exhibit discoloration. In the case of thin samples, the RI and SI algorithms indicated the possibility to detect discoloration on the reverse sides of the measured surfaces. The classification performances of both the RI and SI algorithms using images also surpassed those of the two-waveband ratio and subtraction algorithms using an average spectrum or pixel spectra. The best algorithms in terms of discrimination between discolored and sound areas of lettuce were the RI and SI algorithms. The classification accuracy for lettuce discoloration exhibited by each of these methods using two-waveband spectra and images also surpassed the validation results of image of (680 nm–450 nm)/(680 nm + 450 nm), combination of red and blue wavelengths, obtained using a multispectral vision system with 3-CCD camera designed to detect enzymatic browning in fresh-cut apple slices, which exhibited 84% classification accuracy, as reported by Lunadei *et al.* [32]. These results show that the RI and SI algorithms exhibit the capacity to detect lettuce discoloration.

The results of this study indicate that the combination of two spectral images of the visible range is more suitable than using a single spectral image to detect discolored part in the lettuce. In addition, the combination of two spectral images is possible to detect discolored area on the opposite side of the target surface, which is not possible with a single spectral image. Thus, the spectral information found by the hyperspectral image can be applied to the development of the economic online multispectral imaging system to detect the discoloration of lettuce.

## 4. Conclusions

Nondestructive methods based on visible/near-infrared (VNIR) hyperspectral imaging techniques and employing a single waveband algorithm and multispectral algorithms were developed in order to discriminate between sound and discolored lettuce. The optimal wavebands for discriminating between discolored and sound lettuce surfaces were investigated using the one-way ANOVA method. The multispectral imaging algorithms developed using ratio and subtraction functions resulted in a classification accuracy of above 99.9% for discolored and sound areas on both adaxial and abaxial lettuce surfaces. The R_552/701_ RI algorithm and S_552-701_ SI algorithm using images exhibited better classification performances than in cases where an extraction spectrum such as the average spectrum or pixel spectra were used. The overall results suggest that hyperspectral reflectance imaging techniques have the potential to discriminate between discolored and sound fresh-cut lettuces. In the future, our research will focus on quantitatively predicting the progress of browning on fresh-cut lettuce, in order to improve discrimination accuracy by detecting small defects.
